# Retraction notice for: “lncRNA ZEB1-AS1 inhibits high glucose-induced EMT and fibrogenesis by regulating the miR-216a-5p/BMP7 axis in diabetic nephropathy” [https://doi.org/10.1590/1414-431X20209288]

**DOI:** 10.1590/1414-431X2023e9288retraction

**Published:** 2023-05-15

Qingqing Meng^1^ https://orcid.org/0000-0001-7950-3695, Xiaolin Zhai^1^ https://orcid.org/0000-0001-6572-9941, Yi Yuan^1^ https://orcid.org/0000-0002-9945-1068, Qing Ji^1^ https://orcid.org/0000-0001-6910-042X, and Pengyuan Zhang^1^ https://orcid.org/0000-0001-5913-9152

^1^Department of Nephrology, Luoyang Central Hospital Affiliated to Zhengzhou University, Luoyang, Henan, China

Correspondence: Qingqing Meng: <maipifen3784835@126.com> <mqq18@163.com>

**Retraction for:** Braz J Med Biol Res | doi: 10.1590/1414-431X20209288 | PMCID: PMC7162581 | PMID: 32294702

The Editors of the Brazilian Journal of Medical and Biological Research received a request from the authors to retract the article “lncRNA ZEB1-AS1 inhibits high glucose-induced EMT and fibrogenesis by regulating the miR-216a-5p/BMP7 axis in diabetic nephropathy” that was published in volume 53 no. 4 (2020) (Epub April 9, 2020) of the Brazilian Journal of Medical and Biological Research.

The corresponding author stated: “After careful consideration, we must retract this article, because the data obtained in recent experiments deviated considerably from the data of Figures 2B, 2C, 3E, 3F, 5C, and 5D. We believe that there were errors in the original experiment, resulting in the errors of experimental data. The relationship between ZEB1-AS1, BMP7, and miR216a-5p is uncertain”.

The Editors requested the authors to provide additional information regarding the inconsistencies found in the study. However, the authors presented no further details on that issue. Considering the inaccurate data and experimental deviation detected, the Editors agreed with and endorsed the decision to retract the paper.

The Brazilian Journal of Medical and Biological Research had received authorization from all authors before the publication of the original paper.

